# Reducing Blood Loss in Revision Total Hip and Knee Arthroplasty: Tranexamic Acid Is Effective in Aseptic Revisions and in Second-Stage Reimplantations for Periprosthetic Infection

**DOI:** 10.1155/2018/3891870

**Published:** 2018-11-15

**Authors:** Franz Reichel, Christoph Peter, Volker Ewerbeck, Marcus Egermann

**Affiliations:** ^1^Department of Orthopaedic and Trauma Surgery, University Hospital Heidelberg, Schlierbacher Landstr. 200a, 69118 Heidelberg, Germany; ^2^Department of Anaesthesiology, University Hospital Heidelberg, Im Neuenheimer Feld 110, 69120 Heidelberg, Germany; ^3^Catholic Hospital Mainz, An der Goldgrube 11, 55131 Mainz, Germany

## Abstract

**Introduction:**

The aim of the study was to determine the usefulness of tranexamic acid (TXA) in revision total hip arthroplasty (rTHA) and revision total knee arthroplasty (rTKA). We analyzed the perioperative blood loss with and without TXA in aseptic rTHA and rTKA as well as in second-stage reimplantation for hip and knee periprosthetic infection.

**Materials and Methods:**

In this prospective cohort study, 147 patients receiving TXA (96 rTHA, 51 rTKA) were compared to a retrospective cohort of 155 patients without TXA (103 rTHA, 52 rTKA). The TXA regimen consisted of a preoperative bolus of 10 mg/kg bodyweight (BW) TXA plus 1 mg/kgBW/h perioperatively. Given blood products were documented and the perioperative blood loss was calculated. Thromboembolic events were registered until three months postoperatively. In subgroups, the effects of TXA were separately analyzed in 215 aseptic revisions as well as in 87 reimplantations in two-stage revisions for periprosthetic infection.

**Results:**

Both TXA groups showed a significantly reduced mean blood loss compared to the respective control groups. The TXA group of rTHA patients had a mean blood loss of 2916 ml ± 1226 ml versus 3611 ml ± 1474 ml in the control group (p<.001). For the TXA group of rTKA patients, mean calculated blood loss was 2756 ml ± 975 ml compared to 3441 ml ± 1100 ml in the control group (p=.0012). A significantly reduced blood loss was also found in the TXA subgroups for aseptic and septic revision procedures. No thromboembolic events were recorded among the TXA groups.

**Conclusions:**

There is a significant reduction of perioperative blood loss under TXA influence without an increased incidence of adverse events. The standard use of TXA can be recommended in aseptic hip and knee revision arthroplasties as well as in second-stage reimplantations for periprosthetic infection.

## 1. Introduction

Revision arthroplasty procedures are mostly associated with higher blood loss than primary implantations [1]. Subsequently, there is a greater demand for allogeneic blood transfusion during and after these operations [2]. Even though blood transfusions today are safer than in the past, they are still accommodated by adverse events like allergic reactions or other negative side effects [3]. The transfusion of allogeneic blood products may be associated with adverse patient outcome as well as increased morbidity and mortality [4, 5]. In arthroplasty procedures, blood transfusions have been reported to be a risk factor for periprosthetic infection [6].

For patient safety and economic reasons a variety of methods to minimize the use of blood products have been developed and summarized under the concept of patient blood management [7–9]. Particularly, one of the antifibrinolytic agents, tranexamic acid (TXA), has been studied broadly in recent years. A significant impact on perioperative blood conservation in primary hip and knee arthroplasty without increasing the risk of thromboembolic events has been reported [10–25]. However, there is only minimal literature on the effect and complication rates of TXA in revision procedures, including septic revisions.

Therefore, the purpose of this study was to evaluate if the usage of TXA in revision hip and knee arthroplasty (i) reduces the perioperative blood loss, (ii) lowers the intra- and postoperative transfusion rates, and (iii) does not increase the rate of deep vein thrombosis (DVT) or pulmonal embolism (PE).

## 2. Material and Methods

We performed a prospective cohort study after establishing a standard operating procedure (SOP) for the use of tranexamic acid in our department. Starting in July 2015, every patient undergoing revision total hip arthroplasty (rTHA) or revision total knee arthroplasty (rTKA) received a bolus of 10 mg/kg bodyweight (BW) TXA as well as a continuous dose of 1 mg/kgBW/h intraoperatively. The patients received the bolus prior to skin incision. Up to December 2016, 96 rTHA patients and 51 rTKA patients could be included in this study. The inclusion criteria were patients undergoing any type of aseptic revision of one or more prosthetic components (except isolated liner exchange) or reimplantation in a two-stage procedure for periprosthetic infection. The explantation procedures for periprosthetic infection were not included. Patients with allergy to TXA, a history of thromboembolic events, or DVT/PE were excluded from the study. The inclusion and exclusion criteria were identical in the TXA- and no-TXA-group.

Revision procedures from January 2014 to June 2015, before starting the SOP, were used as retrospective control group. Each prospective cohort of revision patients receiving TXA was compared to a retrospective cohort without TXA. In this manner, 96 prospectively collected rTHA patients with TXA were compared to 103 retrospectively collected rTHA patients without TXA application. Likewise, the prospectively assessed 51 rTKA patients receiving TXA were compared to 52 retrospectively assessed rTKA patients who did not receive TXA.

Subgroup analyses were carried out to examine the effect in aseptic revisions and in reimplantation procedures separately.

From the patients' records, the following parameters were investigated: the operative procedures, the preoperative blood levels of hemoglobin as well as on postoperative days one, three, and five, hematocrit, and creatinine, including hemostasis indicators (international normalized ratio, partial thromboplastin time, antithrombin, and fibrinogen) as well as the operative risk factors like preoperative anemia, history of thromboembolic events, infection, fracture, tumor, and cardiac, renal, or pulmonary dysfunction. The risk factors were represented by the ASA score [26]. Furthermore, given blood products and the postoperative occurrence of complications were registered. The main outcome variables were the calculated blood loss as well as the thromboembolic complications like DVT and PE.

The perioperative blood loss was calculated according to the Brecher formula [27]. Variables required for the computation are the patient's blood volume, the preoperative hematocrit (Ht), the Ht at the postoperative day 5 (POD 5), and all given blood products including intraoperative cell salvage. Patient's blood volume was calculated using height, weight, and gender of the patient [28, 29]. Compared to other methods used for the assessment of perioperative blood loss, the Brecher formula is one of the few methods that take the so-called hidden blood loss into account [27].

The perioperative thrombosis prophylaxis included a daily dose of 40 mg enoxaparin given subcutaneously to all patients for a minimum of 28 days beginning on postoperative day 1. Patients presenting clinical signs for DVT were examined using Doppler ultrasound. Suspected PE were diagnosed or ruled out via CT pulmonary angiography. Any complication was recorded during a follow-up period of three months postoperatively.

All surgical procedures were performed by 10 senior surgeons. Reimplantations in two-stage exchange procedures were performed 6-12 weeks after explantation and antibiotic spacer implantation. If a tourniquet was used in rTKA procedures, it was placed at the level of the upper thigh and inflated to 350 mm Hg prior to cementing the prosthetic components. The tourniquet was deflated after wound closure and application of compression dressing.

Data was collected using the hospital information system. The statistical analyses were performed using SPSS 22 (IBM Corporation, Armonk, NY). Descriptive analysis was carried out and distribution diagrams were used to control for Gaussian distribution. Levene's test assessed the equality of the variances of the given variables.

Student's t-test was used to compare means between the TXA and no-TXA groups if normal distribution and equal variances were present. Welch's test was used if no equal variances were found. Mann-Whitney U test was applied if no normal distribution was encountered. Cross tabulation was used for nominal scaled variables like the complication frequency. For all tests, two-sided significance was assumed for p values below .05. Post hoc computed power analyses for the t-tests of the mean blood loss were carried out for rTHA and rTKA groups (.999 and .912).

The study was approved by the Ethics Committee of the University under the number S-413/2014 and registered at the Federal Institute for Drugs and Medical Devices filed under the number NIS 3377. Therefore, it is in accordance with the ethical standards on human experimentation.

## 3. Results

Between January 2014 and December 2016, a total of 517 rTHA or rTKA were performed at our institution. 215 patients had to be excluded because either patients did not receive TXA according to the protocol or patients received TXA for individual reasons prior to the start of the SOP. The remaining 199 patients undergoing rTHA and 103 patients undergoing rTKA could be included in the study. The preoperatively recorded demographic data and blood variables showed no statistically significant differences between the TXA group and the no-TXA group (Table 1).

For the rTHA group, the most common indication for revision was aseptic loosening of one or more components. Half of the patients presented with this diagnosis. The second most common reason for rTHA was infection or septic loosening with 22% in the TXA-group. 10% of patients were revised because of periprosthetic fracture in the TXA-group and 8% because of hip dislocation. In contrast, the most common indication in the rTKA group was infection beforehand with 41% in the TXA-group, followed by aseptic loosening with 35%. Between the TXA group and the no-TXA group, there were no significant differences concerning the indications for revision surgery (p=.43 for rTHA and p=.25 for rTKA).

Almost one-third of all cases were reimplantations in two-stage revisions for periprosthetic infection. In rTHA and rTKA, the numbers of exchanged components showed no statistically significant differences between the TXA group and the no-TXA group (p=.69 and p=.06, Table 2).

Regarding the use of a tourniquet in the rTKA groups, we found a statistically significant reduced application in the TXA-group with 55% vs 86% in the no-TXA-group (p=.01).

**Figure 1:**
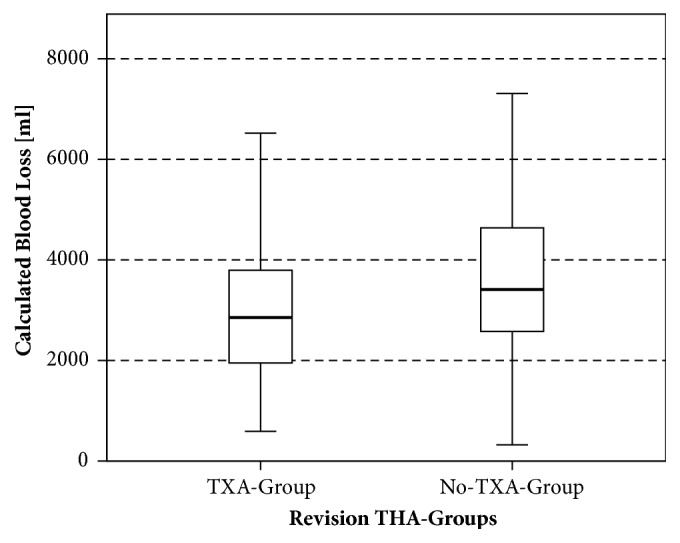
Calculated blood loss of the revision THA groups.

**Figure 2:**
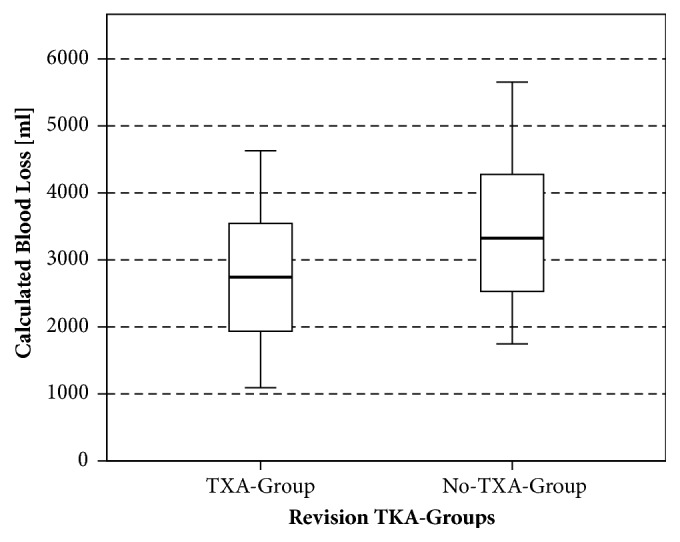
Calculated blood loss of the revision TKA groups.

We found a statistically significant decrease in mean calculated blood loss with the usage of TXA in rTHA and rTKA (Table 3, Figures 1 and 2). In rTHA patients, calculated blood loss was 2916 ml ± 1226 ml with TXA compared to 3611 ml ± 1474 ml without TXA (p<.001). In rTKA patients, a blood loss of 2756 ml ± 975 ml with TXA was calculated compared to 3441 ml ± 1100 ml without TXA (p=.0012). Revision THA patients receiving TXA showed a significant higher Ht on POD 5 (p=.03) as well as a statistically significant lower amount of transfused packed red blood cells (RBC, p=.04) than rTHA patients without TXA.

No thromboembolic events were registered in the no-TXA rTKA group and both TXA groups. One patient undergoing a rTHA without TXA was diagnosed with pulmonal embolism. Therefore, no statistically significant difference regarding the thromboembolic events was found between the TXA and the no-TXA groups.

### 3.1. Subgroup Analysis

Four separate subgroup analyses were carried out to determine if the blood sparing effect of TXA could be registered for aseptic revisions in THA and TKA as well as in hip and knee reimplantations for periprosthetic infection.

The demographic data of all four subgroups showed no significant difference between the TXA and no-TXA groups.

Regarding the aseptic revisions, the blood loss in the TXA groups of aseptic rTHA and aseptic rTKA was significantly decreased (2740 ml ± 1220 ml vs. 3342 ml ± 1304, p<0.01 and 2411 ml ± 979 vs. 3053 ml ± 957, p<0.05). The Ht on POD5 was significantly higher in both TXA groups and the total amount of transfused RBC in the TXA group of the rTHA patients was significantly lower (Table 4).

Regarding the reimplantations in two-stage exchange procedures, the blood loss in the TXA groups of reimplantation THA and TKA was significantly decreased (3544 ml ± 1052 vs. 4882 ml ± 1604, p<0.01 and 3249 ml ± 744 vs. 3801 ml ± 1117, p<0.05; Table 5). No reinfection occurred in the TXA or no-TXA groups within 12 months postoperatively.

## 4. Discussion 

The results of the present study suggest that the use of TXA reduces blood loss in rTHA and rTKA without increasing the risk for thromboembolic events. The use of TXA was effective and safe, regardless of whether aseptic revisions or reimplantations in two-stage exchange procedures for periprosthetic infection were analyzed.

Substantial blood loss is one of the main issues in orthopaedic surgery, leading to an increased complication rate and the need for transfusion [9, 30]. Thus, a variety of methods like the use of TXA have been developed for minimizing blood loss.

In contrast to the abundant literature regarding TXA use in primary THA and TKA, there are only a few studies to this date examining the impact of TXA in either rTHA or rTKA [31–38]. All studies reported a benefit of TXA in revision arthroplasty without an increase in complication rates, but had specific limitations, which the current study tried to surpass. Most of the previous authors excluded reimplantations or revisions for septic loosening. For rTHA, only Kazi et al. included second-stage revision procedures into their study plan with a limited number of six reimplantations in the TXA group and six reimplantations in the control group [31]. In rTKA, Smit et al. presented data in which revision for septic loosening was not excluded, including 57 reimplantations in the TXA group and 24 in the control group [37]. Waddell et al. used a topical administration of TXA before wound closure in 20 patients with infected TKA in the first-stage revision (explantation and antibiotic spacer placement) and in 28 patients in the second-stage revision (reimplantation) [39].

To our knowledge, the current study is the first one which includes aseptic revisions as well as reimplantations in two-stage exchange procedures of THA and TKA. Unlike most of the previous authors, we recorded a high number of cases and excluded only isolated liner exchange procedures because of the expected minor blood loss. With this heterogeneity, our patient cohort reflects the everyday spectrum of a center for revision surgery.

All previous revision arthroplasty studies reported a decrease in blood loss related parameters like hemoglobin drop, transfusion rate, or transfused RBC. Our study can support and strengthen this statement finding that TXA decreased the calculated total blood loss. Moreover, we registered a significant lower amount of transfused RBC as well as a significant higher Ht on POD 5 in the TXA group of rTHA patients. There was a tendency for a decreased transfusion rate and decreased transfused RBC in the TXA group of rTKA patients although not reaching statistically significant difference. The tendency for reduced transfusions and a higher postoperative Ht on POD5 results in the significant statistical difference of calculated blood loss because they are both part of the Brecher calculation formula.

For rTKA only a statistically significant difference for the calculated blood loss and not for transfused RBCs was found. The reduced application of a tourniquet in the TXA-group might have had an influence here. Although the minimum clinically important difference for blood loss is unclear, we believe that every reduction in blood loss is beneficial.

Previous authors except Kazi et al. did not calculate the absolute perioperative blood loss and reported only indirect blood loss related parameters as main endpoints. Kazi et al. used the formula according to Gross et al. but were unable to find a difference between the TXA and the control group for the calculated blood loss in a relatively small number of 60 patients [31, 40]. We determined the overall blood loss of the procedures according to the Brecher formula which includes the hidden blood loss postoperatively and is thought to be an accurate measurement [41–43]. This may be the reason for the slightly higher calculated blood loss of the present study compared to previous publications using different calculating methods [41].

Concerning the postoperative complications, Kazi et al. were the only authors who did find a small increase in thromboembolic events for TXA patients without reaching statistical relevance. However, their small patient collection must be considered where only a few events can have a significant impact. In contrast, the current study can underline the findings of all other authors that no increased thromboembolic complications or complications overall were registered.

Regarding the systemic TXA-regime the present study is in line with most of previous studies. The range of total TXA applicated was between 10 mg/kgBW given by Samujh et al. and 3 g given by Noordin et al., who did not report a strict application regime [34, 36].

There are several limitations in our study. The study was not prospectively randomized; in fact a prospective study group of revision cases receiving TXA was compared to a retrospective one without TXA. A relevant number of patients (215/517) had to be excluded from the present study either because they did not receive TXA according to the protocol due to individual reasons or because TXA was applied prior to the start of the SOP. Septic explantations as the first step of two-stage revision procedures could not be included in our study because TXA was only used in reimplantation procedures during the time of this study.

Revision arthroplasty cohorts are naturally heterogenous. Yet, the preoperative data of our collective showed no difference between the TXA and the no-TXA groups. Even the surgical time, as one of the main indicators for blood loss, was not significantly different between the TXA and no-TXA groups. The large range of surgical times reflects the underlying heterogeneity in revision arthroplasty procedures.

Furthermore, we did not include patients with a history of DVT or PE in our study which might lessen the applicability of the general statement that TXA does not increase the risk for thromboembolic events. To this date, studies including high risk patients are still missing. Due to ethical reasons we could not perform a randomized, controlled trial in our revisions whereas the benefits of TXA in primary arthroplasty surgery as well as in other fields of surgery have been well documented. There are still concerns regarding the safety of TXA application in morbid patients. Surprisingly, data to support these concerns are nonexistent.

## 5. Conclusion

We conclude that TXA is a viable tool to decrease the absolute perioperative blood loss in aseptic revision procedures of THA and TKA as well as in second-stage reimplantations for periprosthetic infection. The use of TXA reduces blood transfusions and does not increase thromboembolic complications. TXA can be recommended as a standard routine for aseptic revisions and reimplantation procedures. Future investigations are warranted to clarify if TXA can also be safely administered to thromboembolic high-risk patients and if so whether or not topical use of TXA may be an alternative.

## Figures and Tables

**Table 1:** Demographic data and preoperative blood variables. Given are mean values (SD), except the absolute amounts for female gender.

**Demographic data**	**TXA** Revision THA n=96 Revision TKA n=51	**No TXA** Revision THA n=103 Revision TKA n=52	**p-value**
Age [years]			
Revision THA	66.1 (13.5)	68.6 (11.3)	0.16^†^
Revision TKA	65.3 (15.2)	66.1 (12.4)	0.78
Female gender, N (%)			
Revision THA	57 (59%)	56 (54%)	0.48
Revision TKA	26 (51%)	28 (54%)	0.77
Height [m]			
Revision THA	1.69 (0.11)	1.69 (0.10)	0.68
Revision TKA	1.70 (0.10)	1.69 (0.11)	0.57
Weight [kg]			
Revision THA	76.6 (16.1)	77.6 (18.6)	0.69
Revision TKA	85.2 (21.2)	88.0 (22.3)	0.51
Calculated blood volume [ml]			
Revision THA	4943 (870)	4865 (877)	0.86
Revision TKA	5205 (948)	5265 (1070)	0.77
ASA score			
Revision THA	2.52 (0.78)	2.48 (0.58)	0.65^†^
Revision TKA	2.47 (0.64)	2.44 (0.57)	0.81
Preoperative Ht			
Revision THA	0.386 (0.051)	0.388 (0.047)	0.71
Revision TKA	0.391 (0.053)	0.391 (0.041)	0.97
INR preop.			
Revision THA	1.01 (0.07)	1.01 (0.07)	0.51
Revision TKA	1.01 (0.06)	1.01 (0.06)	0.71

TXA, tranexamic acid; THA, total hip arthroplasty; TKA, total knee arthroplasty; ASA, American Society of Anesthesiologists; Ht, hematocrit; INR, international normalized ratio; SD, standard deviation; ^†^Welch's test

**Table 2:** Revised components.

**Components being revised**	**TXA** Revision THA n=96 Revision TKA n=51	**No TXA** Revision THA n=103 Revision TKA n=52	**p-value**
**Revision THA**			0.63
Acetabular component	43	43	
Femoral component	22	26	
Both components	10	16	
Reimplantation of both components in two-stage revisions	21	18	0.44

**Revision TKA**			0.06
Femoral component	9	2	
Tibial component	1	3	
Both components	20	20	
Reimplantation of both components in two-stage revisions	21	27	0.28

TXA, tranexamic acid; THA, total hip arthroplasty; TKA, total knee arthroplasty; *∗*: significant; †: Welch's test.

**Table 3:** Main outcome variables. Given are the mean values (SD), in addition to minimum, maximum, and median for surgical time.

**Outcome variables**	**TXA** Revision THA n=96 Revision TKA n=51	**No TXA** Revision THA n=103 Revision TKA n=52	**p-value**
**Revision THA**			
Surgical time [min]	152.6 (51.6)	150.0 (63.7)	0.7500
Min; Max; Median	50; 290; 150	60; 420; 130	
Ht POD 5	0.288 (0.029)	0.278 (0.029)	0.0300*∗*
INR postop.	1.09 (0.07)	1.08 (0.07)	0.4700
RBC postop. [unit]	1.00 (1.11)	1.41 (1.49)	0.0280*∗*^†^
RBC transfused total [unit]	1.49 (1.62)	2.01 (1.92)	0.0400*∗*
Transfusion rate	0.57 (0.50)	0.65 (0.48)	0.2600^†^
Calc. blood loss [ml]	2916 (1226)	3611 (1474)	0.0004*∗*
DVT/PE	0/0	0/1	-/0.3500
Complication rate	0.21 (0.41)	0.15 (0.36)	0.2700^†^
**Revision TKA**			
Surgical time [min]	175.1 (43.5)	174.0 (53.5)	0.9100
Min; Max; Median	105; 300; 165	120; 360; 163	
Ht POD 5	0.279 (0.037)	0.274 (0.025)	0.3800^†^
INR postop.	1.09 (0.07)	1.11 (0.09)	0.2200
RBC postop [unit]	0.78 (1.15)	1.13 (1.37)	0.1600
RBC transfused total [unit]	1.18 (1.57)	1.54 (1.66)	0.2600
Transfusion rate	0.47 (0.50)	0.58 (0.50)	0.2900
Calc. blood loss [ml]	2756 (975)	3441 (1100)	0.0012*∗*
DVT/PE	0/0	0/0	-/-
Complication rate	0.23 (0.43)	0.29 (0.46)	0.5300

TXA, tranexamic acid; THA, total hip arthroplasty; TKA, total knee arthroplasty; POD, postoperative day; Ht, hematocrit; INR, international normalized ratio; RBC, packed red blood cells; DVT, deep vein thrombosis; PE, pulmonal embolism; *∗*: significant; †: Welch's test.

**Table 4:** Main outcome variables of the subgroup analysis between aseptic revisions. Given are the mean values (SD), in addition to minimum, maximum, and median for surgical time.

**Outcome variables**	**TXA** aseptic rTHA n=75 aseptic rTKA n=30	**No TXA** aseptic rTHA n=85 aseptic rTKA n=25	**p-value**
**Aseptic rTHA**			
Surgical time [min]	151.1 (52.7)	140.0 (50.6)	0.1790^†^
Min; Max; Median	50; 290; 150	60; 270; 125	
Ht POD 5	0.290 (0.031)	0.280 (0.029)	0.0453*∗*
RBC transfused total [unit]	1.19 (1.39)	1.73 (1.74)	0.0320*∗*
Transfusion rate	0.51 (0.50)	0.61 (0.49)	0.1833
Calc. blood loss [ml]	2740 (1220)	3342 (1304)	0.0031*∗*
Complication rate	0.24 (0.43)	0.12 (0.33)	0.0550^†^
**Aseptic rTKA**			
Surgical time [min]	163.0 (38.9)	169.0 (46.8)	0.6056
Min; Max; Median	105; 260; 150	120; 255; 165	
Ht POD 5	0.295(0.038)	0.276 (0.025)	0.0290*∗*^†^
RBC transfused total [unit]	1.03 (1.73)	1.20 (1.58)	0.7132
Transfusion rate	0.37 (0.49)	0.48 (0.51)	0.4056
Calc. blood loss [ml]	2411 (979)	3053 (957)	0.0178*∗*
Complication rate	0.25 (0.44)	0.25 (0.44)	1.0000

TXA, tranexamic acid; rTHA, revision total hip arthroplasty; rTKA, revision total knee arthroplasty; POD, postoperative day; Ht, hematocrit; INR, international normalized ratio; RBC, packed red blood cells; DVT, deep vein thrombosis; PE, pulmonal embolism; *∗*: significant; †: Welch's test.

**Table 5:** Main outcome variables of the subgroup analysis between reimplantations. Given are the mean values (SD), in addition to minimum, maximum, and median for surgical time.

**Outcome variables**	**TXA** Reimplantation THA n=21 Reimplantation TKA n=21	**No TXA** Reimplantation THA n=18 Reimplantation TKA n=27	**p-value**
**Reimplantation THA**			
Surgical time [min]	157.9 (48.1)	196.9 (94.1)	0.1239
Min; Max; Median	90; 290; 150	110; 420; 175	
Ht POD 5	0.280 (0.023)	0.268 (0.029)	0.1964
RBC transfused total [unit]	2.57 (1.91)	3.33 (2.22)	0.2571
Transfusion rate	0.81 (0.40)	0.83 (0.38)	0.8517
Calc. blood loss [ml]	3544 (1052)	4882 (1604)	0.0035*∗*
Complication rate	0.10 (0.31)	0.28 (0.46)	0.1173^†^
**Reimplantation TKA**			
Surgical time [min]	192.4 (44.9)	178.6 (59.5)	0.3804
Min; Max; Median	130; 300; 180	120; 360; 160	
Ht POD 5	0.256 (0.020)	0.271 (0.025)	0.0273
RBC transfused total [unit]	1.38 (1.32)	1.85 (1.70)	0.3015
Transfusion rate	0.62 (0.50)	0.67 (0.48)	0.7388
Calc. blood loss [ml]	3249 (744)	3801 (1117)	0.0464*∗*^†^
Complication rate	0.21 (0.41)	0.33 (0.48)	0.3846

TXA, tranexamic acid; THA, total hip arthroplasty; TKA, total knee arthroplasty; ASA, American Society of Anesthesiologists; Ht, hematocrit; INR, international normalized ratio; SD, standard deviation; ^†^Welch's test.

## Data Availability

The original data used to support the findings of this study are available from the corresponding author upon request.

## References

[1] Sculco T. P. (1998). Global blood management in orthopaedic surgery. *Clinical Orthopaedics and Related Research*.

[2] Callaghan J. J., O'Rourke M. R., Liu S. S. (2005). Blood management: issues and options. *The Journal of Arthroplasty*.

[3] Funk M., Lohmann A., Witzenhausen C. (2016). *Haemovigilance report of the Paul-Ehrlich-Institut 2013/2014: Assessment of the reports of serious adverse transfusion reactions pursuant to section 63i AMG (trans: Biomedicines FIfVa)*.

[4] Vamvakas E. C., Blajchman M. A. (2009). Transfusion-related mortality: the ongoing risks of allogeneic blood transfusion and the available strategies for their prevention. *Blood*.

[5] Shander A., Fink A., Javidroozi M. (2011). Appropriateness of allogeneic red blood cell transfusion: The international consensus conference on transfusion outcomes. *Transfusion Medicine Reviews*.

[6] Zhu Y., Zhang F., Chen W., Liu S., Zhang Q., Zhang Y. (2015). Risk factors for periprosthetic joint infection after total joint arthroplasty: A systematic review and meta-analysis. *Journal of Hospital Infection*.

[7] Goodnough L. T., Shander A. (2012). Patient blood management. *Anesthesiology*.

[8] Sambandam B., Batra S., Gupta R., Agrawal N. (2013). Blood conservation strategies in orthopedic surgeries: A review. *Journal of Clinical Orthopaedics and Trauma*.

[9] Levine B. R., Haughom B., Strong B., Hellman M., Frank R. M. (2014). Blood management strategies for total knee arthroplasty. *Journal of the American Academy of Orthopaedic Surgeons*.

[10] Alshryda S., Sarda P., Sukeik M., Nargol A., Blenkinsopp J., Mason J. M. (2011). Tranexamic acid in total knee replacement: a systematic review and meta-analysis. *The Journal of Bone & Joint Surgery—British Volume*.

[11] Cid J., Lozano M. (2005). Tranexamic acid reduces allogeneic red cell transfusions in patients undergoing total knee arthroplasty: results of a meta-analysis of randomized controlled trials. *Transfusion*.

[12] Dunn C. J., Goa K. L. (1999). Tranexamic acid: a review of its use in surgery and other indications. *Drugs*.

[13] Gandhi R., Evans H. M. K., Mahomed S. R., Mahomed N. N. (2013). Tranexamic acid and the reduction of blood loss in total knee and hip arthroplasty: A meta-analysis. *BMC Research Notes*.

[14] Gill J. B., Rosenstein A. (2006). The use of antifibrinolytic agents in total hip arthroplasty: a meta-analysis. *The Journal of Arthroplasty*.

[15] Huang F., Wu D., Ma G., Yin Z., Wang Q. (2014). The use of tranexamic acid to reduce blood loss and transfusion in major orthopedic surgery: A meta-analysis. *Journal of Surgical Research*.

[16] Kim T. K., Chang C. B., Koh I. J. (2014). Practical issues for the use of tranexamic acid in total knee arthroplasty: A systematic review. *Knee Surgery, Sports Traumatology, Arthroscopy*.

[17] Melvin J. S., Stryker L. S., Sierra R. J. (2015). Tranexamic Acid in Hip and Knee Arthroplasty. *Journal of the American Academy of Orthopaedic Surgeons*.

[18] Shemshaki H., Nourian S. M. A., Nourian N., Dehghani M., Mokhtari M., Mazoochian F. (2015). One step closer to sparing total blood loss and transfusion rate in total knee arthroplasty: a meta-analysis of different methods of tranexamic acid administration. *Archives of Orthopaedic and Trauma Surgery*.

[19] Sukeik M., Alshryda S., Haddad F. S., Mason J. M. (2011). Systematic review and meta-analysis of the use of tranexamic acid in total hip replacement. *The Journal of Bone and Joint Surgery—British Volume*.

[20] Wang C., Xu G.-J., Han Z. (2015). Topical application of tranexamic acid in primary total hip arthroplasty: A systemic review and meta-analysis. *International Journal of Surgery*.

[21] Wei Z., Liu M. (2015). The effectiveness and safety of tranexamic acid in total hip or knee arthroplasty: A meta-analysis of 2720 cases. *Transfusion Medicine*.

[22] Yang Z.-G., Chen W.-P., Wu L.-D. (2012). Effectiveness and safety of tranexamic acid in reducing blood loss in total knee arthroplasty: A meta-analysis. *The Journal of Bone & Joint Surgery*.

[23] Zhang H., Chen J., Chen F., Que W. (2012). The effect of tranexamic acid on blood loss and use of blood products in total knee arthroplasty: a meta-analysis. *Knee Surgery, Sports Traumatology, Arthroscopy*.

[24] Fillingham Y. A., Ramkumar D. B., Jevsevar D. S. (2018). The Efficacy of Tranexamic Acid in Total Knee Arthroplasty: A Network Meta-Analysis. *The Journal of Arthroplasty*.

[25] Gianakos A. L., Hurley E. T., Haring R. S., Yoon R. S., Liporace F. A. (2018). Reduction of Blood Loss by Tranexamic Acid Following Total Hip and Knee Arthroplasty. *JBJS Reviews*.

[26] Daabiss M. (2011). American society of anaesthesiologists physical status classification. *Indian Journal of Anaesthesia*.

[27] Brecher M. E., Monk T., Goodnough L. T. (1997). A standardized method for calculating blood loss. *Transfusion*.

[28] Feldschuh J., Enson Y. (1977). Prediction of the normal blood volume. Relation of blood volume to body habitus. *Circulation*.

[29] Feldschuh J., Katz S. (2007). The Importance of Correct Norms in Blood Volume Measurement. *The American Journal of the Medical Sciences*.

[30] Lee C., Freeman R., Edmondson M., Rogers B. A. (2015). The efficacy of tranexamic acid in hip hemiarthroplasty surgery: An observational cohort study. *Injury*.

[31] Kazi H. A., Fountain J. R., Thomas T. G., Carroll F. A. (2012). The effect of bolus administration of tranexamic acid in revision hip arthroplasty. *Hip International*.

[32] Ortega-Andreu M., Talavera G., Padilla-Eguiluz N. G. (2016). Tranexamic Acid in a Multimodal Blood Loss Prevention Protocol to Decrease Blood Loss in Revision Total Knee Arthroplasty: A Cohort Study. *The Open Orthopaedics Journal *.

[33] Park K. J., Couch C. G., Edwards P. K., Siegel E. R., Mears S. C., Barnes C. L. (2016). Tranexamic Acid Reduces Blood Transfusions in Revision Total Hip Arthroplasty. *The Journal of Arthroplasty*.

[34] Noordin S., Waters T. S., Garbuz D. S., Duncan C. P., Masri B. A. (2011). Tranexamic acid reduces allogenic transfusion in revision hip arthroplasty. *Clinical Orthopaedics and Related Research*.

[35] Phillips S. J., Chavan R., Porter M. L. (2006). Does salvage and tranexamic acid reduce the need for blood transfusion in revision hip surgery?. *The Journal of Bone & Joint Surgery (British Volume)*.

[36] Samujh C. (2014). Decreased blood transfusion following revision total knee arthroplasty using tranexamic acid. *The Journal of Arthroplasty*.

[37] Smit K. M., Naudie D. D. R., Ralley F. E., Berta D. M., Howard J. L. (2013). One dose of tranexamic acid is safe and effective in revision knee arthroplasty. *The Journal of Arthroplasty*.

[38] Aguilera X., Videla S., Almenara M., Fernandez J. A., Gich I., Celaya F. (2012). Effectiveness of Tranexamic Acid in revision total knee arthroplasty. *Acta Orthopædica Belgica*.

[39] Waddell B. S., Zahoor T., Meyer M., Ochsner L., Chimento G. (2016). Topical Tranexamic Acid Use in Knee Periprosthetic Joint Infection Is Safe and Effective. *Journal of Knee Surgery*.

[40] Gross J. B. (1983). Estimating allowable blood loss: Corrected for dilution. *Anesthesiology*.

[41] Gibon E., Courpied J.-P., Hamadouche M. (2013). Total joint replacement and blood loss: What is the best equation?. *International Orthopaedics*.

[42] Sehat K., Evans R., Newman J. (2000). How much blood is really lost in total knee arthroplasty?. *The Knee*.

[43] Liu X., Zhang X., Chen Y., Wang Q., Jiang Y., Zeng B. (2011). Hidden Blood Loss After Total Hip Arthroplasty. *The Journal of Arthroplasty*.

